# Taxifolin Inhibits Invasion and Endovascular Differentiation of Extravillous Trophoblast HTR-8/SVneo Cells

**DOI:** 10.3390/cells15131152

**Published:** 2026-06-24

**Authors:** Aleksandra Vilotić, Janko Legner, Žanka Bojić-Trbojević, Marija Bruić, Biljana Spremo-Potparević, Milica Jovanović Krivokuća, Andrea Pirković

**Affiliations:** 1 Department for Biology of Reproduction, Institute for the Application of Nuclear Energy (INEP), University of Belgrade, 11080 Belgrade, Serbia; janko.legner@inep.co.rs (J.L.); zana@inep.co.rs (Ž.B.-T.); andrea.pirkovic@inep.co.rs (A.P.); 2 Department of Pathobiology, Faculty of Pharmacy, University of Belgrade, 11221 Belgrade, Serbia; marija.bruic@pharmacy.bg.ac.rs (M.B.); biljana.potparevic@pharmacy.bg.ac.rs (B.S.-P.)

**Keywords:** taxifolin, extravillous trophoblast, trophoblast invasion, endovascular differentiation, epithelial–mesenchymal transition

## Abstract

Adequate placental development and function, prerequisites for the development of a healthy fetus, rely on controlled trophoblast invasion into the decidua and remodeling of the spiral arteries. These tightly regulated processes involve epithelial–mesenchymal transition (EMT) and endovascular differentiation of trophoblast cells. Taxifolin (dihydroquercetin), a natural flavonoid with various pharmacological effects, previously showed cytoprotective, antioxidant, and anti-inflammatory activity on trophoblast cells. Given that the literature indicates that this flavonoid suppresses EMT and can affect angiogenesis across different cell types, we investigated the potential of taxifolin (10 and 100 µM) to modulate invasion and endothelial-like differentiation in human extravillous trophoblast HTR-8/SVneo cells by functional tests. Expression of different molecular markers relevant to these processes was evaluated at the mRNA and protein levels. Our results showed that taxifolin inhibited invasion of HTR-8/SVneo cells, involving downregulation of integrin α5 subunit and modulation of MMP-2 and MMP-9 mRNA expression and secretion. No changes in the concentrations of secreted TIMP-1 and TIMP-2 were observed following taxifolin treatment. Furthermore, downregulation of N-cadherin and vimentin in treated trophoblast cells indicated suppression of EMT. Taxifolin inhibited endothelial-like differentiation of HTR-8/SVneo cells, as evidenced by reduced tube formation and downregulation of VE-cadherin in treated cells. Moreover, expression of *TGFB1* was upregulated in treated cells, as were levels of phosphorylated SMAD2/3, indicating involvement of TGF-β signaling in TF-induced effects on trophoblast cells. The in vitro effects of taxifolin on suppression of trophoblast invasion, EMT, and endothelial-like differentiation highlight its potential impact on placental development processes.

## 1. Introduction

A healthy pregnancy is dependent on adequate development and function of the placenta. Trophoblast cells have a key role in these processes, especially during the initial stages of pregnancy, when the feto-maternal interface is being established. During implantation, the outer cell layer of the blastocyst, trophectoderm, differentiates into the early trophoblast lineages, which further differentiate into several distinct trophoblast cell subtypes with specific roles in placentation and placental function [1,2]. Cytotrophoblast cells (CTBs), mononucleated progenitor trophoblast cells, continuously divide and, by fusion, give rise to the multinucleated syncytiotrophoblast (STB). STB covers the chorionic villi and is in direct contact with maternal blood, enabling the transport of substances between the mother and the fetus, i.e., nutrients, gases, and waste products [2]. Through the process of epithelial–mesenchymal transition (EMT), CTBs at the tips of the anchoring villi differentiate into invasive extravillous trophoblast cells (EVTs). Interstitial EVTs invade uterine tissue, infiltrating decidual stroma and part of myometrium, anchoring the placenta to the uterus while endovascular EVTs migrate intraluminally along the wall of decidual spiral arteries and together with interstitial EVTs remodel walls of these vessels, transforming them into wide conduits non-responsive to the vasomotor control, enabling adequate blood supply to the placenta and fetus throughout gestation [2,3].

CTBs undergoing EMT go through substantial phenotypic changes, including loss of cell polarity, alteration in cytoskeleton organization, cell–cell and cell–matrix interactions, and gain of migratory and invasive capacity [4,5]. These phenotypic changes are a result of expression modulation of various molecular mediators, with a loss of epithelial and a gain of mesenchymal markers. Characteristic repertoire “switch” of cell adhesion molecules, including integrins, which mediate cell–matrix interactions, and cadherins, which mediate cell–cell adhesion, takes place during the differentiation of CTBs to an invasive phenotype [5,6]. “Integrin switching” is represented by downregulation of α6β4 integrin characteristic for epithelial CTBs and upregulation of α5β1 and α1β1 integrins in differentiating trophoblast and EVTs [6,7]. Characteristic features of EMT include upregulation of mesenchymal markers such as neuronal-cadherin (N-cadherin) and vimentin [8]. The important role of N-cadherin for trophoblast invasion and interaction with endothelial cells is documented in the literature [9,10,11,12,13] as well as upregulation of vimentin in EVTs [14]. Matrix metalloproteinase (MMP)-2 and MMP-9 are important mediators of trophoblast invasion [15]. These enzymes facilitate the degradation of the extracellular matrix (ECM) and enable trophoblast cells to invade decidual tissue. Tissue inhibitors of matrix metalloproteinases (TIMPs) suppress the activity of MMPs and are thus involved in the regulation of trophoblast invasion, limiting its extent [15]. Balanced secretion of MMPs and TIMPs at the feto-maternal interface is important for adequate trophoblast invasion.

During endovascular migration and remodeling of spiral arteries, EVTs acquire endothelial-like properties, including expression of cell adhesion molecules characteristic of endothelial cells [16,17]. Endovascular differentiation enables interaction of trophoblast cells with endothelial cells, integration into the endothelium, and transient replacement of the vessel lining [18]. Invasion of trophoblast cells into the decidua and remodeling of the spiral arteries are processes of utmost importance for the establishment of the feto-maternal interface, the utero-placental circulation, and the efficient functioning of the placenta, which are prerequisites for the development of a healthy fetus. Disruption of these processes leads to the development of different pregnancy complications, such as preeclampsia, intrauterine growth restriction, late miscarriage, and others [19,20,21].

Taxifolin (TF), also known as dihydroquercetin, is a bioactive flavonoid with various pharmacological effects [22,23,24]. It can be extracted from coniferous trees, including Siberian and Dahurian larch trees, Douglas fir bark, and French maritime pine [22]. It is present in different plants used in traditional medicine [25,26,27,28] as well as in different commercially available health-promoting products used as food additives and dietary supplements [27,29,30,31,32]. Furthermore, TF can be found in foods and beverages widely used in the human diet, such as onions [33], olive oil [34], citrus fruits [35], apples [36], berries [37,38], grapes, wine, and green tea [39]. Numerous studies have reported beneficial effects of TF on human health, including antioxidant [40], anti-inflammatory [41], hepatoprotective [42], and anticancer activities [24]. Our previous research showed that TF exhibits cytoprotective, antioxidant, and anti-inflammatory activity in trophoblast cells under oxidative stress, suggesting potential beneficial use of this flavonoid as a protective agent in oxidative stress-related pregnancy complications [43,44]. Nevertheless, there is not enough data on the potential effects of TF during pregnancy, especially on early pregnancy events. Moreover, the literature shows that TF suppresses EMT and invasion in different cancer cells [45,46,47,48,49,50] and can potentially affect angiogenesis [51,52,53], but the effects of TF on trophoblast cells in this context have not yet been studied. The aim of this research was to investigate the potential of TF to affect trophoblast function. We evaluated trophoblast invasion and endothelial-like differentiation of human EVT HTR-8/SVneo cells after exposure to TF, as well as expression of relevant mediators of these processes.

## 2. Materials and Methods

### 2.1. Cell Culture

The human HTR-8/SVneo EVT cell line was obtained by the courtesy of Dr. Charles H. Graham (Queen’s University, Kingston, ON, Canada) [54]. This cell line reproduces key phenotypic and functional characteristics of human primary trophoblast cells, including invasive and migratory properties, expression of key trophoblast markers, and responds to different experimental conditions in a manner comparable to the primary EVT cells [54,55,56]. The HTR-8/SVneo cells were maintained in RPMI 1640 medium (Biowest, Nuaillé, France) supplemented with 10% heat inactivated newborn calf serum (FCS) (Biowest, Nuaillé, France) and 1% antibiotic/antimycotic solution (PAN-Biotech, Aidenbach, Germany), referred to hereafter as complete medium. The cells were cultured in a humidified incubator with 5% CO_2_ at 37 °C.

### 2.2. Treatment Preparation

A stock solution (100 mM) of TF (Cas No. 480-18-2, Sigma–Aldrich, St. Louis, MO, USA) was made by dissolving powdered TF in dimethyl sulfoxide (DMSO, Carl Roth, Karlsruhe, Germany). For the treatment of the HTR-8/SVneo cells in the experiments, the stock solution was diluted in complete medium to a final concentration of 10 or 100 μM. The cells in the control group were treated with DMSO alone. The concentration of DMSO was set to 0.1% in all conditions. In our previous research [43], it was shown that the TF treatment in chosen concentrations did not affect the viability of HTR-8/SVneo cells, while it showed a cytoprotective effect under oxidative stress.

### 2.3. Spheroid Invasion Assay

Spheroid invasion assay in Matrigel was used to assess how TF affects the invasion abilities of HTR-8/SVneo cells. Spheroids were made by a hanging-drop method using 5000 cells per 25 μL drop of complete medium. After 96 h in culture, spheroids were treated with 10 or 100 μM TF for 24 h in hanging drops. After treatment, spheroids were transferred to wells of 96-well plates, one spheroid per well, embedded in Matrigel (2.5 mg/mL, 40 μL per well), and left for 30 min at 37 °C to allow Matrigel to solidify. Next, 100 μL of complete medium was added to the wells, and images of spheroids were captured at 0 h (T0) and after 24 h (T24) using a 4× objective on an Olympus IX73 inverted microscope equipped with an Olympus SC50 digital camera (Olympus, Tokyo, Japan). Spheroid areas at T0 and invaded areas at T24 were measured using ImageJ 1.53t software (National Institutes of Health, Bethesda, MD, USA). The relative invasion rate was quantified by normalizing T24-invaded areas to the corresponding T0 spheroid areas (T24/T0). The mean value of the relative invasion rate for the controls was set to 100%, and the experimental data are presented as percentages of control values. The experiments were performed four times, with three to seven replicates per experiment.

### 2.4. Tube Formation Assay

The tube formation assay was done as previously described [11]. Briefly, HTR-8/SVeo cells were seeded in 12-well plates (3 × 10^5^ cells/well) and left overnight to attach. The next day, cells were treated with 10 or 100 μM TF in complete medium for 24 h. After incubation, cells were detached from the plates using 0.25% trypsin-EDTA solution, and 4 × 10^4^ cells/well were seeded in Matrigel-coated wells of a 96-well plate. We used growth factor-reduced Matrigel in a final concentration of 7 mg/mL. After 5 h of incubation, images of tubes were taken at 4× magnification using an Olympus IX73 inverted microscope equipped with the Olympus SC50 digital camera (Olympus, Tokyo, Japan). Four non-overlapping fields of view per well were analyzed. Total tube length and number of branching points were quantified using ImageJ 1.53t software (National Institutes of Health, USA). The branching point was considered a point from which two or more tubes branched. The mean value for the control was set to 100%, and data are presented as a percentage of the control. The experiments were repeated three times in duplicates.

### 2.5. Quantitative PCR Analysis

HTR-8/SVneo cells were seeded in 24-well plates at 1.2 × 10^5^ cells/well and incubated overnight to attach. The next day, cells were incubated with 10 or 100 μM TF in complete medium for 24 h. After treatment, conditioned media were collected for further analysis by ELISA test (Section 2.8), and cells were rinsed once in PBS and lysed in TRI reagent solution. Total RNA was isolated by phase separation and alcohol precipitation according to the manufacturer’s instructions. For cDNA synthesis, 1 μg of total RNA was used, and the reaction mixture included 0.5 μg of Oligo(dT) 12–18 primers, 250 μM of each dNTP, and 200 U of RevertAid reverse transcriptase. All reagents listed above were from Thermo Fisher Scientific Baltics, Vilnius, Lithuania. The acquired cDNA was diluted 2× in DNase-free water. The qPCR reaction mixture contained 1 μL of diluted cDNA, 5 μL of SG/ROX qPCR Master Mix (EURx Ltd., Gdańsk, Poland), specific forward and reverse primers (Table 1) in a final concentration of 0.5 μM, and DNase-free water. The following temperature profile was used for qPCR reactions programmed on the Applied Biosystems 7500 Real-Time PCR System (Thermo Fisher Scientific, Waltham, MA, USA): 95 °C for 10 min for initial denaturation and enzyme activation, followed by 40 cycles of denaturation for 15 s at 95 °C and primer annealing with elongation for 1 min at 60 °C. Amplification specificity was assessed by melting curve analysis. The housekeeping gene *ACTB* was used to normalize the expression levels of *ITGA1*, *ITGA5*, *ITGB1*, *MMP2*, *MMP9*, *TIMP1*, *TIMP2*, *CDH2* (N-cadherin), *CDH5* (VE-cadherin), *VIM*, and *TGFB1*. Relative gene expression was calculated by the 2^−ΔΔCt^ method [57]. The experiments were performed at least three times in one to three replicates.

### 2.6. Cell-Based ELISA (cELISA)

Cells were seeded in 96-well plates (2 × 10^4^ cells/well), allowed to attach overnight, and then treated with 10 or 100 μM TF in complete medium for 24 h. After incubation, the medium was discarded, and cells were rinsed with PBS, fixed in 4% paraformaldehyde for 20 min at room temperature (RT), and permeabilized with 0.1% of Triton X-100 (10 min, RT) for labeling cells with antibodies specific to integrins. For labeling with antibodies specific to N-cadherin and vimentin, cells were dried after rinsing with PBS and fixed with ice-cold acetone/methanol (1:1) for 5 min, dried, and rehydrated with PBS before the next steps. The endogenous peroxidase activity was blocked with 0.3% H_2_O_2_ in the dark (30 min, RT). After rinsing with PBS and blocking with 1% bovine serum albumin (BSA) (30 min, RT), the cells were incubated with primary antibodies diluted in 1% BSA in a humidified chamber overnight at 4 °C. We used mouse anti-human integrin α1 (MAB5676, R&D Systems, Minneapolis, MN, USA), rabbit anti-human integrin α5 (sc-10729, Santa Cruz Biotechnology, Santa Cruz, CA, USA), rabbit anti-human integrin β1 (AB1952, Merck KGaA, Darmstadt, Germany), rabbit anti-human N-cadherin (orb501008, Biorbyt Ltd., Cambridge, UK), or rabbit anti-human vimentin (#5741, Cell Signaling Technology, Danvers, MA, USA) antibodies. For the assessment of non-specific binding of secondary antibodies, the cells were incubated in 1% BSA only. After incubation with primary antibodies, the cells were rinsed with PBS containing 0.05% Tween-20 (5 × 5 min), and HRP-linked secondary antibodies (horse anti-mouse IgG (#7076, Cell Signaling Technology, USA) or goat anti-rabbit IgG (#7074, Cell Signaling Technology, USA)) were added to the cells. Following a 1 h incubation at RT in the dark and rinsing cells with PBS, 25 μL/well of substrate (0.3% H_2_O_2_) and 25 μL/well of chromogen (0.05% 3,3′,5,5′-Tetramethylbenzidine (TMB)) were added and incubated in the dark. The reaction was stopped with 0.2 M H_2_SO_4_ (50 μL/well). The absorbance was read at 450 nm on a microplate reader (Epoch, BioTek, Winooski, VT, USA). After subtracting the absorbance for non-specific binding from all absorbances, the mean value for the control cells was set to 100%, and the data were presented as a percentage of the control value. All experiments were repeated at least three times in three to five replicates.

### 2.7. Gelatine Zymography

Substrate in gel zymography was used to evaluate the secretion of MMP-2 and MMP-9 by treated HTR-8/SVneo cells. The cells were seeded in complete medium (3 × 10^5^ cells/well; 24-well plates) and the next day, after rinsing with PBS, 10 or 100 μM TF in serum-free RPMI medium was added to the cells. After 24 h incubation, the conditioned media were collected, centrifuged at 300× *g*, and the supernatants were used for analysis. The Pierce bicinchoninic acid protein assay (Thermo Fisher Scientific, Rockford, IL, USA) was used to determine protein concentration in the samples. Equal amounts of protein per sample were subjected to electrophoresis under non-reducing conditions using 11% SDS-polyacrylamide gel containing 1 mg/mL of gelatin as previously described [67]. The ChemiDoc Imaging System (Bio-Rad Laboratories, Singapore) was used for imaging zymograms, and gelatinolytic activity was semi-quantified by densitometric analysis using the ImageJ 1.53t software (National Institutes of Health, USA). The mean value for the control samples was set to 100%, and the data were presented as a percentage of control values. The experiment was conducted three times in one or two replicates.

### 2.8. Western Blot Analysis

HTR-8/SVneo cells were seeded in a 24-well plate at 2 × 10^4^ cells per well and left to adhere overnight. The following day, cells were rinsed, and the treatment (TF at 10 or 100 μM) incomplete medium (for p-SMAD and SMAD detection) or in serum-free medium (for MMP detection) was added. The cells were incubated for 6 h (for p-SMAD detection) or 24 h (for MMP detection). HTR-8/SVneo whole cell lysates were analyzed for p-SMAD2/3 and SMAD3 protein expression, while conditioned media were analyzed for MMP-2 and MMP-9 protein expression. Samples were prepared in 0.125 M Tris-HCl buffer, containing 4% SDS, 20% glycerol, 0.1% bromphenol blue, and 10% 2-mercaptoethanol by heating at 100 °C for 10 min. Proteins were separated by SDS-polyacrylamide gel electrophoresis on a 10% polyacrylamide gel. Following electrophoresis, proteins were transferred to a nitrocellulose membrane. Non-specific binding was blocked by 1% BSA in PBS. The following primary antibodies were used: mouse anti-human p-SMAD2/3 (sc-11769, Santa Cruz Biotechnology, USA), mouse anti-human SMAD3 (sc-101154, Santa Cruz Biotechnology, USA), rabbit anti-human MMP-2 (sc-10736, Santa Cruz Biotechnology, USA), and rabbit anti-human MMP-9 (sc-10737, Santa Cruz Biotechnology, USA). After overnight incubation with primary antibodies at 4 °C, the membranes were rinsed and incubated with secondary antibodies for 1 h at RT (HRP-linked horse anti-mouse IgG (#7076, Cell Signaling Technology, USA) or goat anti-rabbit IgG (#7074, Cell Signaling Technology, USA)). For SMAD3 detection, after staining for p-SMAD2/3, membranes were stripped and re-probed with the primary antibody. Proteins were detected with Pierce ECL Western Blotting Substrate (Thermo Fisher Scientific, Rockford, IL, USA). The obtained signals were scanned and analyzed by the ImageMaster TotalLab v2.01 program (Amersham Biosciences, Inc., Piscataway, NJ, USA). The intensity of protein bands for p-SMAD2/3 was normalized to total SMAD3 and then expressed as a percentage of the untreated control. The results for MMP-2 and MMP-9 secreted levels were presented as a percentage of the untreated control. The experiments were conducted at least three times.

### 2.9. ELISA Test

The conditioned media were collected after treatment (Section 2.5) and stored at −20 °C until the analysis. The evaluation of TIMP-1 and TIMP-2 levels was assessed by using colorimetric sandwich ELISA kits according to the manufacturer’s instructions with the Human TIMP1 ELISA kit (ab187394, Abcam, Cambridge, UK) and the Human TIMP2 ELISA Kit (ab270213, Abcam, Cambridge, UK). Briefly, 50 μL of each standard and sample was added to the appropriate wells, followed by the addition of 50 μL of antibody cocktail (detector and capture antibody mixture), and the mixture was incubated at RT for 1 h with gentle shaking (400 rpm). After washing three times, 100 μL of TMB development solution was added to all wells and incubated for 10 min in the dark at RT with gentle shaking (400 rpm). Next, the stop solution (100 μL/well) was added to all wells, followed by the measurement of absorbance at 450 nm in a UV/Vis microplate reader (VICTOR 1420 Multilabel Counter, Perkin Elmer, Shelton, CT, USA). The experiment was performed three times, with at least two replicates per trial.

### 2.10. Statistical Analysis

Differences between experimental groups were analyzed using one-way analysis of variance (ANOVA) because the data were normally distributed, followed by a Tukey’s post hoc test (α = 0.05) using GraphPad Prism 11.0.2 (GraphPad Software, Inc., La Jolla, SD, USA). Values were considered to be significantly different when *p* < 0.05.

## 3. Results

### 3.1. Taxifolin Inhibits Invasion of HTR-8/SVneo Cells

**Figure 1:**
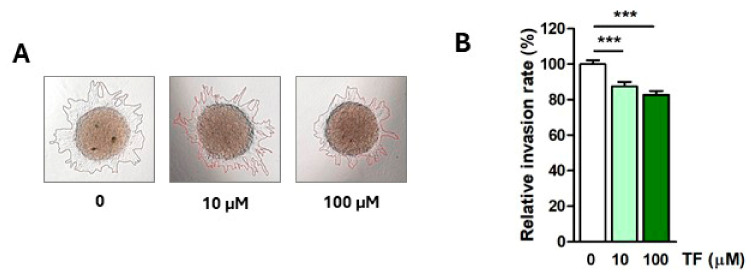
The effect of taxifolin (TF) on the invasion of HTR-8/SVneo cells. (**A**) Representative images of the spheroid invasion assay. Images were taken 24 h after embedding spheroids in Matrigel. (**B**) Relative invasion rate of treated HTR-8/SVneo spheroids with 10 or 100 µM TF for 24 h. Data are presented as mean + SEM of four experiments (n = 4). One-way ANOVA with Tukey’s post hoc test was used to detect significant differences among the three experimental groups. *** *p* < 0.001.

To investigate the effects of TF on human trophoblast invasion, HTR-8/SVneo spheroids were treated with 10 or 100 µM TF for 24 h. Since we were interested in investigating the direct effect of TF on trophoblast cells and wanted to avoid potential interactions between TF and Matrigel, the treatment was performed in hanging drops before spheroids were embedded in Matrigel. The spheroid invasion assay showed that TF in both administered concentrations significantly inhibited invasion of HTR-8/SVneo cells, namely the relative invasion rate was decreased to 87.5 ± 2.5% (10 μM TF; *p* < 0.001) and 82.8 ± 2.1% (100 μM TF; *p* < 0.001) compared to the control (Figure 1).

### 3.2. Taxifolin Inhibits Endothelial-like Differentiation of HTR-8/SVneo Cells Involving VE-Cadherin Downregulation

**Figure 2:**
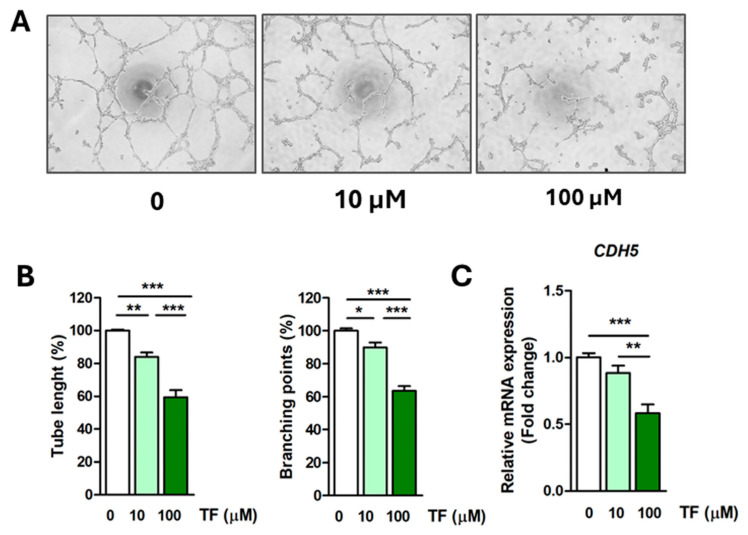
The effect of taxifolin (TF) on tube formation and VE-cadherin expression in HTR-8/SVneo cells. (**A**) Representative images of HTR-8/SVneo tube formation assay on Matrigel. The images were acquired 5 h after seeding cells on Matrigel. (**B**) Tube length and number of branching points made by HTR-8/SVneo cells treated with 10 or 100 µM TF for 24 h. (**C**) Relative mRNA expression of *CDH5* in HTR-8/SVneo cells treated with 10 or 100 µM TF for 24 h. Data are presented as mean + SEM of two ((**B**), n = 3) or four ((**C**), n = 4) experiments. One-way ANOVA with Tukey’s post hoc test was used to detect significant differences among the three experimental groups. * *p* < 0.05, ** *p* < 0.01, *** *p* < 0.001.

Tube formation assay on Matrigel was used to assess the ability of HTR-8/SVneo cells to acquire an endothelial-like phenotype. TF significantly inhibited tube formation of HTR-8/SVneo cells in a concentration-dependent manner (Figure 2A). TF induced a decrease in tube length to 84.1 ± 2.6% for 10 μM (*p* < 0.01) and 59.5 ± 4.3% for 100 μM TF (*p* < 0.001), while branching points were decreased after treatment with 10 μM TF to 90 ± 3% (*p* < 0.05) and after 100 μM TF to 63.7 ± 2.8% (*p* < 0.001) compared to control (Figure 2B). Furthermore, our results showed downregulation of VE-cadherin (*CDH5*) mRNA levels in HTR-8/SVneo cells after treatment with 100 µM TF (fold change 0.58 ± 0.06, *p* < 0.001), indicating that TF affects tube formation by regulating VE-cadherin expression.

### 3.3. Taxifolin Downregulates Expression of Integrin α5 Subunit

**Figure 3:**
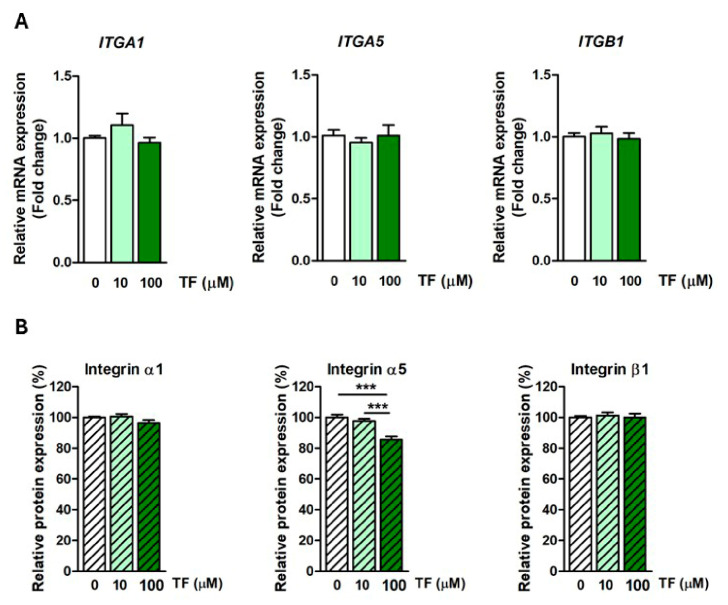
Relative expression of integrin subunits α1 (*ITGA1*), α5 (*ITGA5*), and β1 (*ITGB1*) at mRNA (**A**) and protein (**B**) levels in HTR-8/SVneo cells treated with taxifolin (TF). HTR-8/SVneo cells were treated with 10 or 100 μM TF for 24 h. Data are presented as mean + SEM. The experiments were repeated 3 to 4 times (n = 3–4). One-way ANOVA with Tukey’s post hoc test was used for statistical analysis. *** *p* < 0.001.

Since TF inhibited HTR-8/SVneo invasion and tube formation, in order to further elucidate its possible mechanism of action, we investigated the expression of molecular markers relevant to these processes in cells exposed to TF. We evaluated expression of integrin subunits α1, α5, and β1 at mRNA and protein levels using qPCR and cELISA test, respectively. Higher concentration of TF, 100 µM, significantly downregulated protein expression of integrin α5 subunit to 85.5 ± 2.1% (*p* < 0.001) in treated cells without affecting its mRNA level (Figure 3) compared to the control group. TF did not affect the expression of α1 or β1 integrin subunits at either the mRNA or protein level (Figure 3).

### 3.4. Taxifolin Modulates Expression and Secretion of MMP-2, MMP-9, TIMP-1, and TIMP-2

**Figure 4:**
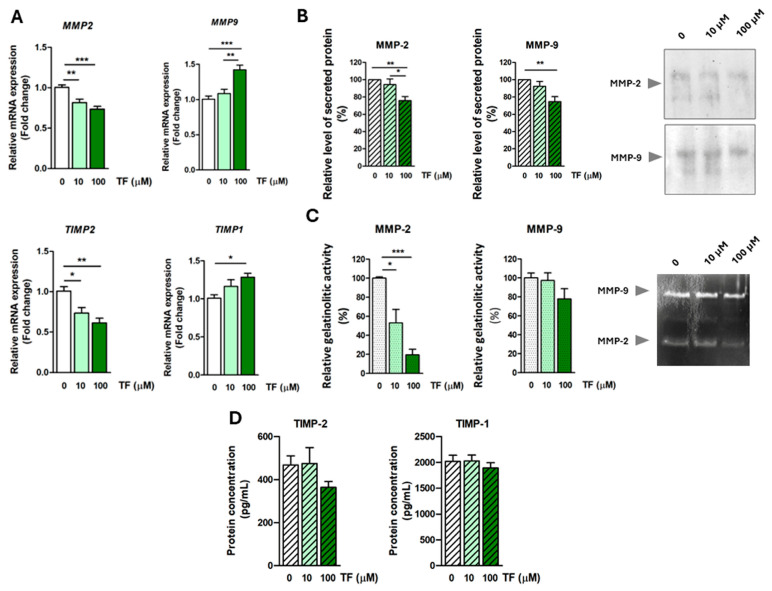
The effect of taxifolin (TF) on the expression and secretion of MMP-2, MMP-9, TIMP-2, and TIMP-1 in HTR-8/SVneo cells treated with 10 or 100 µM TF for 24 h. (**A**) Relative mRNA expression of *MMP2*, *MMP9*, *TIMP2*, and *TIMP1* in treated HTR-8/SVneo cells evaluated by qPCR. (**B**) Relative level of secreted MMP-2 and MMP-9 in conditioned media of treated HTR-8/SVneo cells assessed by Western blot. (**C**) Relative gelatinolytic activity of MMP-2 and MMP-9 in conditioned media of treated HTR-8/SVneo cells assessed by gelatin zymography and representative zymogram. (**D**) Concentration of TIMP-2 and TIMP-1 in conditioned media of treated HTR-8/SVneo cells assessed by ELISA test. Data are presented as mean + SEM of five ((**A**), *MMP2*, *MMP9*, *TIMP1*; (**B**), MMP-2 n = 5), four ((**A**), *TIMP2* n = 4) or three ((**B**) MMP-9, (**C**,**D**) n = 3) experiments. One-way ANOVA with Tukey’s post hoc test was used to detect significant differences among the three experimental groups. * *p* < 0.05, ** *p* < 0.01, *** *p* < 0.001.

Trophoblast invasion is regulated by the coordinated activity of proteolytic enzymes, which degrade ECM, such as MMPs, and inhibitors that regulate their activity, such as TIMPs. We evaluated expression of MMP-2 and MMP-9, important mediators of trophoblast invasion, and their respective inhibitors, TIMP-2 and TIMP-1, in cells treated with TF at the mRNA level by qPCR. TF treatment induced downregulation of *MMP2* and *TIMP2* (*MMP2*—fold change 0.82 ± 0.04, *p* < 0.01 for 10 µM, 0.74 ± 0.03, *p* < 0.001 for 100 µM; *TIMP2*—fold change 0.74 ± 0.07, *p* < 0.05 for 10 µM, 0.61 ± 0.06, *p* < 0.01 for 100 µM TF) while *MMP9* and *TIMP1* were upregulated in treated cells (*MMP9*—fold change 1.42 ± 0.07, *p* < 0.001 for 100 µM TF; *TIMP1*—fold change 1.28 ± 0.05, *p* < 0.05 for 100 µM TF) compared to control (Figure 4A). Further, the level of secreted MMP-2 and -9 in conditioned media of HTR-8/SVneo cells assessed by Western blot was decreased after treatment with 100 µM TF compared to control (MMP-2—75.9 ± 4.6%, *p* < 0.01; MMP-9—74.7 ± 5.7, *p* < 0.01) (Figure 4B). Gelatin zymography showed that the gelatinolytic activity of MMP-2 in conditioned media of treated cells was significantly decreased after treatment with TF (to 53 ± 14.3%, *p* < 0.05 for 10 µM and to 19.5 ± 5.8%, *p* < 0.001 for 100 µM TF compared to control), while the activity of secreted MMP-9 did not significantly change after TF treatment, although a decrease could be noticed at higher TF concentration (100 µM) (Figure 4C). No change in TIMP-1 and -2 concentration in conditioned media of HTR-8/SVneo cells was observed following TF treatment, although a decrease in secreted TIMP-2 at 100 µM TF treatment could be noticed (Figure 4D).

### 3.5. Taxifolin Modulates Expression of EMT Markers

**Figure 5:**
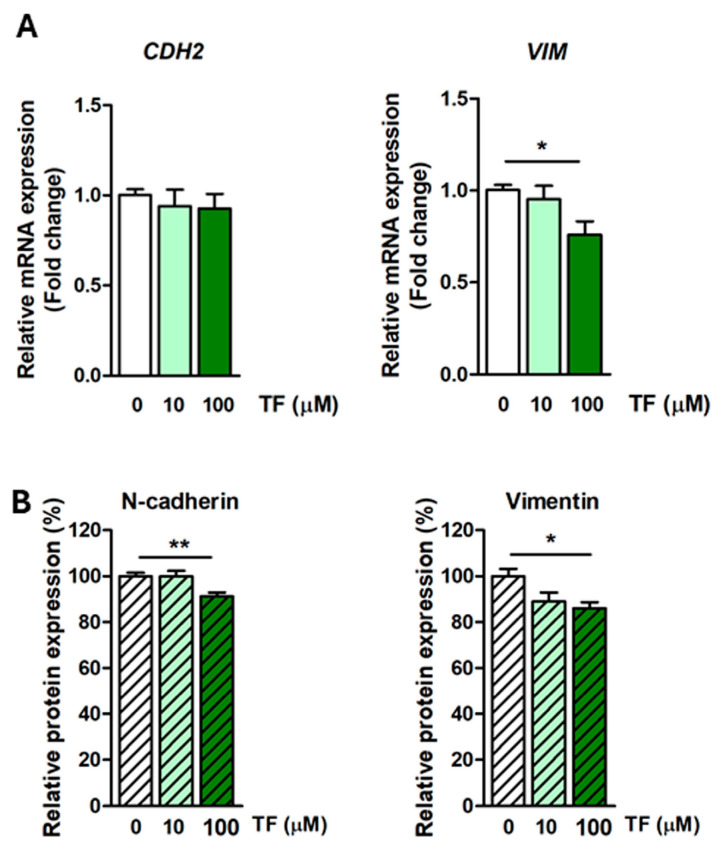
Relative expression of N-cadherin (*CDH2*), and vimentin (*VIM*) at mRNA (**A**) and protein (**B**) levels in HTR-8/SVneo cells treated with taxifolin (TF). HTR-8/SVneo cells were treated with 10 or 100 μM TF for 24 h. Data are presented as mean + SEM of three (Vimentin, n = 3), five (*CDH2*, *VIM* n = 5), or six (N-cadherin, n = 6) experiments. One-way ANOVA with Tukey’s post hoc test was used to detect significant differences among the three experimental groups. * *p* < 0.05, ** *p* < 0.01.

CTB cells undergo EMT during differentiation into EVT, gaining migratory and invasion capacity. This process includes upregulation of mesenchymal markers, including N-cadherin (*CDH2*) and vimentin [8]. Treatment with a higher concentration of TF (100 µM) downregulated mRNA and protein expression of vimentin (mRNA fold change 0.76 ± 0.07, *p* < 0.05; protein—86.2 ± 2.6%, *p* < 0.05 compared to the control) as well as protein expression of N-cadherin (91.3 ± 1.6%, *p* < 0.01 compared to control) (Figure 5). These results indicate that TF could modulate EMT in trophoblast cells.

### 3.6. Taxifolin Activates TGF-β Signaling Pathway

**Figure 6:**
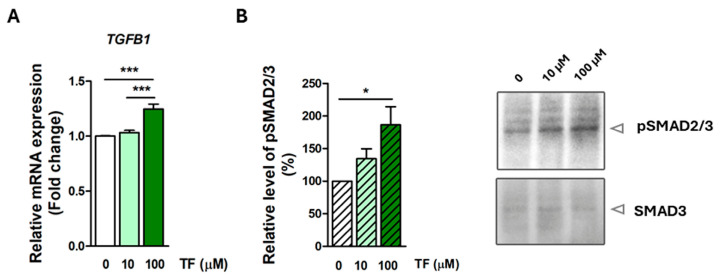
Relative expression of *TGFB1* assessed by qPCR (**A**) and relative level of phosphorylated SMAD2/3 (pSMAD2/3) assessed by Western blot (**B**) in HTR-8/SVneo cells treated with taxifolin (TF). The cells were treated with 10 or 100 μM TF for 24 h (**A**) or 6 h (**B**). Data are presented as mean + SEM of four (A n = 4) or five (B n = 5) experiments. One-way ANOVA with Tukey’s post hoc test was used for statistical analysis. * *p* < 0.05, *** *p* < 0.001.

TGF-β signaling plays an important role in the regulation of trophoblast function, including differentiation and invasion [68,69]. TGF-β influences the expression of EMT-associated transcription factors, cell adhesion molecules, and ECM-remodeling enzymes. Effects of TF treatment on TGF-β signaling in HTR-8/SVneo cells were evaluated by assessment of *TGFB1* mRNA expression by qPCR and evaluation of TGF-β signaling pathway activation through assessment of SMAD2/3 phosphorylation level by Western blot. *TGFB1* was upregulated (fold change 1.25 ± 0.04, *p* < 0.001 compared to control) (Figure 6A) and the level of p-SMAD2/3 was increased (to 186.6 ± 27.6%, *p* < 0.05 compared to control) (Figure 6B) in HTR-8/SVneo cells treated with 100 µM TF. These results indicate involvement of the TGF-β signaling in TF-induced effects on trophoblast cells.

## 4. Discussion

TF is well known for its various biological effects and potential benefits for human health [22,23,24]. Although TF is used as a dietary supplement in the general population, there is no clinical evidence in humans supporting its safety or efficacy during pregnancy. While some animal studies and in vitro data suggest its protective effects against oxidative stress–related damage in reproductive tissues, data on pregnancy effects are limited and inconclusive [70,71,72]. In our previous research on trophoblast cells under oxidative stress, TF showed cytoprotective, antioxidant, and anti-inflammatory effects, indicating its potential beneficial use in pregnancy complications connected to oxidative stress [43,44]. However, further research is necessary to elucidate the potential effects of TF on early pregnancy events and pregnancy as a whole.

In this study, we used the human EVT HTR-8/SVneo cell line as a model system for studying the effects of TF on processes important for early pregnancy and placentation. Our previous research showed that treatment with TF up to 100 µM concentration did not affect HTR-8/SVneo cell viability, while it exerted a protective effect against oxidative stress [43]. In the present study, 10 µM TF was chosen to approximate the upper range of potentially achievable plasma/tissue exposure following TF-enriched supplementation, whereas 100 µM was used as a supraphysiological/pharmacological concentration that had previously demonstrated no cytotoxicity in vitro.

Proper development and function of the placenta, which is necessary for pregnancy success, is largely dependent on adequate EVT invasion and remodeling of spiral arteries. EVTs acquire an invasive phenotype through the process of EMT, which is characterized by changes in the expression of different molecular markers, leading to the loss of epithelial and gain of mesenchymal features. Literature data show that TF inhibits the invasion of different mouse and human cancer cells, such as osteosarcoma [45], melanoma [49], breast [46], gastric [48] and lung cancer [47] affecting the expression of MMP-2 and MMP-9 [48,49] among other EMT markers [46,47,48,50]. Our results are in line with the literature data. Using the spheroid invasion assay, we determined that TF inhibited the invasion of HTR-8/SVneo cells in a concentration-dependent manner. To explore the underlying mechanisms of TF effects on trophoblast invasion, we evaluated the expression of different molecular mediators of this process as well as different EMT markers.

Levels of secreted MMP-2 and MMP-9 were significantly lower after treatment of HTR-8/SVneo cells with 100 µM TF compared to untreated cells. TF treatment also negatively affected *MMP2* mRNA expression and gelatinolytic activity of secreted MMP-2. On the other hand, *MMP9* mRNA expression was upregulated after treatment with 100 µM TF, although a decrease in the gelatinolytic activity of this enzyme in the conditioned media of treated cells was observed, but it was not statistically significant. Activity of MMPs and consequently ECM degradation and adequate trophoblast invasion depend on balanced secretion of MMPs and their endogenous inhibitors, TIMPs, at the feto-maternal interface [15]. TIMP-1 has high binding affinity to MMP-9, while TIMP-2 preferentially regulates the activity of MMP-2 [73]. TF modulated mRNA expression of both TIMPs, namely, *TIMP1* expression was upregulated, while *TIMP2* was downregulated. On the other hand, levels of secreted TIMPs were not significantly changed by TF treatment. According to these results, it can be concluded that TF-induced inhibition of invasion involved a reduction in MMP-2 and -9 secretion.

Modulation of mRNA expression is not always followed by corresponding changes in protein abundance [74,75]. Gene expression is regulated at different post-transcriptional levels, including alternative splicing, regulation of mRNA stability, non-coding RNA-mediated transcription/translation regulation, and others [74]. Furthermore, protein expression levels could also be affected by the modulation of protein stability and degradation rates. These mechanisms could explain discrepancies between TF-induced changes in mRNA and protein expression.

Furthermore, TF treatment modulated the expression of characteristic EMT markers in HTR-8/SVneo cells. “Cadherin switch”, presented by downregulation of E-cadherin and upregulation of N-cadherin, is a characteristic feature of the EMT process [8]. Vimentin, a major intermediate filament protein in mesenchymal cells, is typically upregulated during the EMT [8]. Treatment with a higher concentration of TF (100 µM) downregulated N-cadherin protein expression as well as vimentin mRNA and protein expression in treated HTR-8/SVneo cells. These results indicate that TF could suppress EMT of trophoblast cells, which could further elucidate the underlying mechanism of TF-induced trophoblast invasion inhibition. A similar effect of TF on EMT markers was shown in different human cancer cells [46,47,48,50]. Downregulation of N-cadherin in HTR-8/SVneo cells by small interfering RNAs (siRNA) or using function-perturbing N-cadherin antibodies inhibited the invasion of these cells [9,12,13]. Moreover, downregulated expression of N-cadherin in trophoblast cells was associated with preeclampsia, a pregnancy complication linked to shallow trophoblast invasion and inadequate transformation of spiral arteries [76,77].

Integrins, adhesion molecules which mediate the interaction of cells with their environment [78] are important mediators of trophoblast invasion [7]. These heterodimeric transmembrane molecules consist of non-covalently bound α and β subunits, which combine into receptors for ECM ligands predominantly [78]. Differentiation of trophoblast cells to the invasive phenotype involves a change in the integrin repertoire, i.e., “integrin switch” [6]. Invasive EVTs have upregulated expression of α5β1 and α1β1 integrins, while α6β4 integrin, characteristic of epithelial cytotrophoblast, is downregulated [6]. Our results showed that 100 µM TF downregulated protein expression of α5 integrin subunit in treated HTR-8/SVneo cells, which is in line with the TF-induced invasion inhibition. Expression of the α5 integrin subunit at the mRNA level in treated cells did not change, which indicates that TF treatment could affect some of the post-transcriptional/translational mechanisms of protein homeostasis. Interestingly, in the study of Kumar et al. TF was identified by bioinformatical methods as a potential inhibitor of α5β1 integrin [79]. It was suggested that TF binds to the ligand-binding pocket of α5β1, exhibiting inhibitory activity on this integrin [79]. This finding remains to be experimentally confirmed, but, together with our results, it indicates that TF could have a dual effect on α5β1 integrin, involving both downregulation of the α5 subunit and inhibition of α5β1 integrin activity, which could contribute to the inhibitory effect of TF on trophoblast invasion.

Remodeling of the uterine spiral arteries is one of the crucial processes important for adequate development of the fetus and maintaining a healthy pregnancy [18]. During the first trimester of pregnancy, EVTs invade and transform the walls of spiral arteries [18]. Due to the degradation of the smooth muscle layer and loss of elastic lamina of the vessel walls, spiral arteries become large, dilated conduits, which ensure high blood flow to the placenta, enabling adequate nutrient supply to the developing fetus [18]. During the remodeling of spiral arteries, endovascular EVTs acquire endothelial-like properties, including expression of cell adhesion proteins characteristic of the endothelial cells, such as VE-cadherin, VCAM-1, PECAM-1, and others [16,80] and transiently replace the endothelium of the spiral arteries [18]. An in vitro assay for assessment of the endothelial-like differentiation of the EVTs is a tube formation assay on Matrigel, i.e., an in vitro angiogenesis assay, in which trophoblast cells, similarly to endothelial cells, form capillary-like structures. Our study showed that treatment with TF inhibited the tube-formation ability of treated HTR-8/SVneo cells in a concentration-dependent manner, indicating that this flavonoid could affect endovascular differentiation of EVTs and, consequently, affect remodeling of the spiral arteries. Further analysis showed that expression of VE-cadherin (*CDH5*) mRNA was downregulated after TF treatment, revealing at least partially the underlying mechanism of the inhibitory effect of TF on tube formation in treated trophoblast cells. VE-cadherin is an adhesion protein specific for endothelial cells, where it plays an essential role in the formation and regulation of endothelial junctions’ stability [81]. Different studies confirmed that VE-cadherin is also expressed in endovascular EVTs, and it was suggested that it enables interaction of trophoblast and endothelial cells during the remodeling of the spiral arteries [16,80]. The important role of trophoblast VE-cadherin in the remodeling of spiral arteries was further emphasized by the in vivo study on mice, where trophoblast-specific deletion of VE-cadherin caused impaired spiral artery remodeling, inhibited the displacement of the endothelium, and degradation of the smooth muscle layer of the walls of these vessels, which caused impaired placentation, fetal growth restriction, and mid-gestation embryonic lethality [82]. Furthermore, literature data show that VE-cadherin also participates in trophoblast invasion [16,83]. Namely, downregulation of VE-cadherin expression by siRNA in primary cytotrophoblast and HTR-8/SVneo cells or using function-perturbing anti-VE-cadherin antibodies inhibited the invasion abilities of these cells in vitro [16,83]. Taking into consideration literature data, it can be concluded that TF-induced downregulation of VE-cadherin contributes to both inhibition of tube formation and decreased invasion of treated HTR-8/SVneo cells.

TGF-β1 signaling plays an important role in placental development and trophoblast function [84] through regulation of adhesion, proliferation, differentiation, migration, and invasion of trophoblast cells [68,69,83,85,86]. Different studies have shown that both exogenous and endogenous TGF-β1 have a negative effect on trophoblast invasion [68,83]. Inhibition of endogenous TGF-β1 by neutralizing antibodies increased the invasion capacity of EVTs [68] while autocrine TGF-β1 signaling suppressed the mesenchymal phenotype of EVTs and negatively affected their migration [69]. TGF-β1-induced inhibition of trophoblast invasion was at least partially mediated by downregulation of VE-cadherin [83]. Our results are in line with the literature data. Namely, our study showed that treatment with 100 µM TF upregulated *TGFB1* mRNA in HTR-8/SVneo cells, which further elucidated the underlying mechanism of TF-induced negative effects on the function of HTR-8/SVneo cells. Canonical TGF-β signaling is mediated through phosphorylation of SMAD2 and SMAD3, followed by the formation of the SMAD2/3–SMAD4 complex, which translocates to the nucleus and regulates gene expression [87]. Increased levels of p-SMAD2/3 could be detected in HTR-8/SVneo cells after treatment with 100 µM TF, indicating involvement of TGF-β signaling in TF-induced effects on trophoblast cells. Based on the literature and the results of our study, it can be proposed that TF-induced inhibition of invasion and tube formation, as well as modulation of EMT, is at least partially mediated by upregulation of TGF-β1 signaling, leading to downregulation of VE-cadherin in treated HTR-8/SVneo cells.

Given the central role of extravillous trophoblast invasion, endovascular differentiation, and spiral artery remodeling in establishing adequate uteroplacental circulation, the observed inhibitory effects of TF on these processes may have implications for placental development and pregnancy complications associated with impaired trophoblast function, including preeclampsia and fetal growth restriction. Although TF is generally regarded as a safe dietary flavonoid, with favorable toxicological profiles reported in animal studies and no major safety concerns identified by the European Food Safety Authority for its use as a food ingredient, available safety data are not specific to pregnancy and placental development [31]. Moreover, recent human studies have reported good tolerability and an absence of adverse events following prolonged TF supplementation [88]. Nevertheless, our findings indicate that special caution should be exercised regarding TF use during early pregnancy, as modulation of key trophoblast functions may adversely affect placental establishment despite its otherwise favorable safety profile.

## 5. Conclusions

The present study investigated the effects of bioactive flavonoid TF on human EVTs from the first trimester of pregnancy. Given that proper EVT function, including adequate differentiation, invasion into the maternal decidua, and remodeling of the spiral arteries, is critical for normal pregnancy progression, our findings provide important insight into the potential impact of TF on these key processes. Our results showed that TF inhibited invasion and endovascular differentiation, and that it modified EMT of EVTs. These effects were mediated by modulation of MMP-2, MMP-9, α5 integrin, and VE-cadherin, as well as EMT markers N-cadherin and vimentin. Furthermore, TGF-β signaling was implicated in TF-induced modulation of trophoblast function. Although further research on physiologically more relevant model systems is necessary, the current in vitro study implies that TF has the potential to affect the function of EVTs and consequently to negatively affect early pregnancy events.

## Figures and Tables

**Table 1:** The sequences of primers used for qPCR.

Gene	Primer Sequence	Reference
*ITGA1*	Forward: 5′-GGTTCCTACTTTGGCAGTATT-3′	[58]
	Reverse: 5′-AACCTTGTCTGATTGAGAGCA-3′	
*ITGA5*	Forward: 5′-GGCAGCTATGGCGTCCCACTGTGG-3′	[59]
	Reverse: 5′-GGCATCAGAGGTGGCTGGAGGCTT-3′	
*ITGB1*	Forward: 5′-GTGGTTGCTGGAATTGTTCTTATT-3′	[59]
	Reverse: 5′-TTTTCCCTCATACTTCGGATTGAC-3′	
*MMP2*	Forward: 5′-TGCGACCACAGCCAACTACG-3′	[60]
	Reverse: 5′-ACAGACGGAAGTTCTTGGTGTAGG-3′	
*MMP9*	Forward: 5′-TGACAGCGACAAGAAGTG-3′	[60]
	Reverse: 5′-CAGTGAAGCGGTACATAGG-3′	
*TIMP1*	Forward: 5′-CATCCTGTTGTTGCTGTGGCTGAT-3′	[61]
	Reverse: 5′-GTCATCTTGATCTCATAACGCTGG-3′	
*TIMP2*	Forward: 5′-CTCGCTGGACGTTGGAGGAAAGAA-3′	[61]
	Reverse: 5′-AGCCATCTGGTACCTGTGGTTCA-3′	
*CDH2*	Forward: 5′-CATCAACCGGCTTAATGGTG-3′	[62]
(N-cadherin)	Reverse: 5′-ACTTTCACACGCAGGATGGA-3′	
*CDH5*	Forward: 5′-GAAGCCTCTGATTGGCACAGTG-3′	[63]
(VE-cadherin)	Reverse: 5′-TTTTGTGACTCGGAAGAACTGGC-3′	
*VIM*	Forward: 5′-AGGCAAAGCAGGAGTCCACTGA-3′	[64]
	Reverse: 5′-ATCTGGCGTTCCAGGGACTCAT-3′	
*TGFB1*	Forward: 5′-ACCAACTATTGCTTCAGCTC-3′	[65]
	Reverse: 5′-TTATGCTGGTTGTACAGG-3′	
*ACTB*	Forward: 5′-AGAGCTACGAGCTGCCTGAC-3′	[66]
	Reverse: 5′-AGCACTGTGTTGGCGTACAG-3′	

## Data Availability

The original contributions presented in this study are included in the article. Further inquiries can be directed to the corresponding authors.
